# A spark of hope: histopathological and functional recovery after critical COVID-19

**DOI:** 10.1007/s15010-021-01678-7

**Published:** 2021-08-25

**Authors:** Anna Boehm, Anna K. Luger, Katja Schmitz, Katharina Cima, Daniel Hölbling Patscheider, Florian Augustin, Lisa Maria Jakob, Astrid Obermayer, Guenter Weiss, Walter Stoiber, Gerlig Widmann, Judith Loeffler-Ragg

**Affiliations:** 1 Department of Internal Medicine II, Infectious Diseases, Pneumology, Rheumatology, Medical University of Innsbruck, Anichstraße 35, 6020 Innsbruck, Austria; 2 Department of Radiology, Medical University of Innsbruck, Innsbruck, Austria; 3 Innpath GmbH, Innsbruck, Austria; 4 Department of Pneumology, Tirol Kliniken, Hospital Hochzirl-Natters, Natters, Austria; 5 Department of Anaesthesia and Critical Care Medicine, Hospital of Brixen, Brixen, Italy; 6 Department of Visceral, Transplant and Thoracic Surgery, Center of Operative Medicine, Medical University of Innsbruck, Innsbruck, Austria; 7 Department of Biosciences, University of Salzburg, Salzburg, Austria

**Keywords:** COVID-19, Long COVID, Recovery, Long-term sequelae

## Abstract

**BACKGROUND:**

There are substantial concerns about fibrotic and vascular pulmonary sequelae after coronavirus disease 2019 (COVID-19) associated acute respiratory distress syndrome (ARDS).AQ1 Histopathology reports of lung biopsies from COVID-19 survivors are scarce.

**CASE:**

We herein report results of functional and histopathological studies in a 70 year-old man undergoing a co-incidental tumor lobectomy six months after long-term mechanical ventilation for COVID-19 pneumonia.

**CONCLUSION:**

Despite several unfavorable risk factors, this case presentation shows a completed pulmonary recovery process within a few months.

## Background

The novel severe acute respiratory syndrome coronavirus 2 (SARS-CoV-2) is known for its potential to cause severe pulmonary damage. Several post-mortem studies on histopathological features of severe coronavirus disease 2019 (COVID-19) have been conducted so far. However, hardly any case reports on the histopathology in COVID-19 survivors exist, describing primarily organizing pneumonia [1–4].

We therefore aimed at investigating the functional and histopathological recovery of a patient with the indication for tumor lobectomy six months after severe SARS-CoV-2 infection.

## Case presentation

On 15 March 2020, a 70 year-old man was admitted to a peripheral hospital in South Tyrol (Italy) because of fever (up to 39 °C) and a slightly dry cough. He presented with a history of arterial hypertension, a status post resection of a caecal tubular adenoma in 2018 and a nicotine abuse of 30 pack years until 2010. Nevertheless, the patient was in a good state of health, having run a half marathon two weeks prior to the admission. The performed SARS-CoV-2 test (RT-PCR) showed a positive result. On admission an empiric antimicrobial therapy was initiated with ceftriaxon 2g i.v. once daily and after two days escalated to levofloxacin 750 mg i.v. once daily and amoxicilline-clavulate 2.2 g i.v. tid. As an antiviral therapy the patient received hydroxychloroquine 200 mg p.o. for 8 days and darunavir 800 mg p.o. in combination with ritonavir 100 mg p.o. for 7 days. Remdesivir had been neither available nor recommended by the local treatment protocol at that time in Italy.

**Fig. 1:**
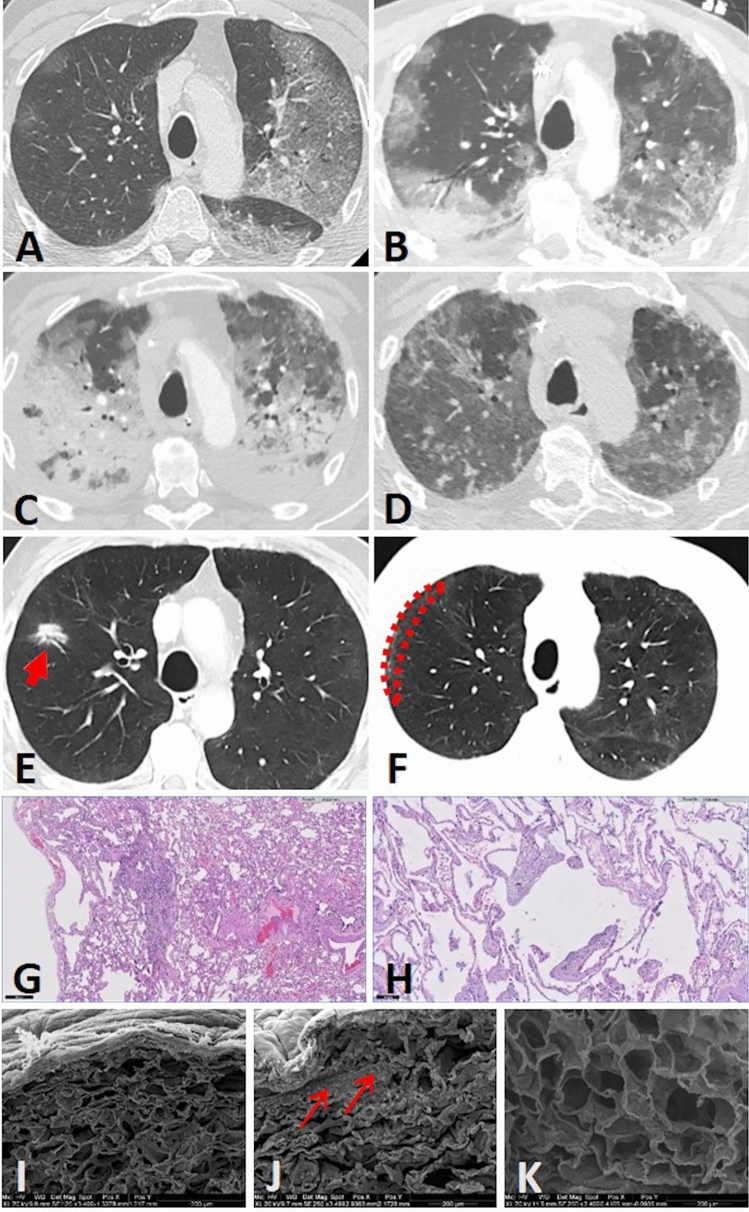
**A**–**F** Serial computed tomography (CT) scans in axial slices. **A** was the earliest acquired image 8 days after proven severe acute respiratory syndrome coronavirus 2 (SARS-CoV2)-infection which showed extensive homogeneous ground-glass changes in the left upper and lower lobe, as well as subtle patchy ground-glass opacities sub-pleural in the right upper lobe. **B** was performed 18 days later showing mixed areas of consolidation and ground-glass opacities on the left side and worsening of patchy ground-glass opacities as well as new consolidations in the dependent lung right sided. **C** was performed another 18 days later due to clinical deterioration. There were now extensive areas of consolidations in both lungs exhibited in accordance with acute respiratory distress syndrome (ARDS). **D** is a control CT after 40 days. Density of former consolidations had decreased markedly. **E** Axial contrast enhanced CT shows a spiculated nodule in the right upper lobe (arrow), on a CT level  < 1 cm below the areas shown in **A–D** + **F,** five months past positive SARS-CoV-2 polymerase chain reaction (PCR). **F** Mild bilaterally residual diffuse ground-glass opacities and sub-pleural reticulations also five months after critical COVID-19. The sub-pleural region of the right upper lobe was histopathologically further processed (circled). **G**, **H** Microscopically sub-pleural thickened alveolar septa and interstitial fibrosis surrounded by preserved alveolar architecture and emphysematous changes. **G** (1819_2) 20 × magnification, **H** (1816_2) 100 × magnification. **I, K** Representative scanning electron microscope (SEM) images. Sub-pleural parenchyma (**I** 125 × magnification**, J** 250 × magnification) exhibits braid-like islands of fibrosis (arrows in **J**), peripheral architectural deformation reveals to be a preparative artifact due to manual touch during surgery and pathological processing; peripheral alveoli are extent thin-walled like those in the inconspicuous deeper parenchyma (**K** 250 × magnification)

**Fig. 2:**
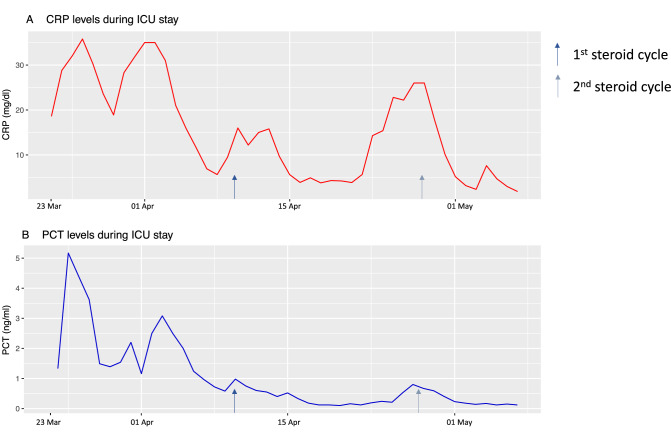
**A** depicts the C-reactive protein (CRP, red) and **B** the pro-calcitonin (PCT, blue) levels during ICU stay. The arrows show the two starting time points of the steroid cycles, the exact treatment regime is mentioned in the main text. The maximum of ferritin was measured on 24 March 2020 at 6317 ng/l, while the maximum interleukin 6 was measured on 28 March 2020 with 254 pg/ml (data not shown)

The following week, the patient’s condition deteriorated rapidly, and on 23 March 2020, intubation and mechanical ventilation with the transfer to the intensive care unit (ICU) were inevitable. For gaining antimicrobial probes the antibiotic therapy was discontinued for 1 day and reinitiated with piperacillin-tazobactam 4.5 g i.v. tid, which was administrated for 11 days. Due to rapidly increasing inflammatory parameters the antibiotic therapy was switched to meropenem and linezolid. Despite, the patient developed an acute respiratory distress syndrome (ARDS) and  ventilationtherapy required a FiO_2_ max of 1.0 for two days, a *P*_insp_ between 20 and 30 mbar and a positive end-expiratory pressure level between 8 and 14 mbar. The patient underwent a total of 12 cycles of pronation with mostly a good response and an overall median increase of the pO_2_ of 50 mmHg. An extubation attempt two weeks later failed. Consecutively, due to the pronounced radiological findings (Fig. 1A–D) and the expected prolonged weaning time, a tracheostomy was performed on 18 May 2020. Of note, because of the lack of experience and evidence no initial corticoid therapy had been started. In the further course the patient received twice a steroid cycle therapy, the first on 9 April 2020 after the failed extubation attempt in the 4th week of his hospitalisation, the second was started on 27 April 2020, in the 7th week (Fig. 2). Firstly, due to a sepsis 200 mg hydrocortisone were administered which was then switched to methylprednisolone for tapering and secondly, 300 mg hydrocortisone was given for 3 days, with consecutive 240 mg methylprednisolone i.v. for 5 days as a rescue therapy because of the poor clinical improvement, which was again tapered for 4 days. Upon the methylprednisolone therapy the patient improved gradually. After approximately 6 weeks of intubation, the weaning phase was successfully concluded on 6 May 2020 and the patient was able to breath spontaneously without any mechanical support.

Overall, the laboratory findings confirmed a status of hyper-inflammation with increased interleukin 6, ferritin, C-reactive protein and pro-calcitonin levels (Fig. 2). The microbiological investigations revealed a moderate growth of *Candida albicans* in the bronchial aspirate without treatment indication from probes that were gained at a timepoint in April, at which the patient was in a stable clinical health status.

Fatefully, in addition to COVID-19, an incidental pulmonary nodule of about 2.5 cm was detected in the right upper lobe in the former smoker. After the patient’s recovery and pulmonary rehabilitation, further clinical diagnostics of the pulmonary nodule revealed the diagnosis of a pulmonary adenocarcinoma. Astonishingly, six months post COVID-19, the 70 year-old man presented with normal spirometry findings, a VO_2_max of 26 ml/min/kg and a normal lung perfusion scintigraphy. Thus, a video-assisted thoracic surgery (VATS) lobectomy of the right upper lobe could be performed on 1 October 2020.

The histopathological evaluation of the tumor-distant-lung parenchyma (craniolateral, Fig. 1F) revealed neither residual signs of late-stage diffuse alveolar damage, nor microvascular alterations, nor signs of interstitial lung disease.

In particular, we were interested in a potential histological correlation to the residual ground-glass opacities and sub-pleural lines (Fig. 1F). In this context, some discrete sub-pleural septal thickening and peri-bronchiolar fibrosis were described histopathologically. Furthermore, electron microscopy confirmed sub-pleural braid-like islands of fibrosis, but otherwise no inter-alveolar septal fibrosis (Fig. I-K). According to the pathologists, peri-bronchiolar fibrosis was rather seen as a smoking-associated sequelae. In consideration of the patient’s good general condition, these changes did not appear to be linked to clinically significant functional impairment.

## Discussion

We herein present a patient, who regained high respiratory functionality after six months despite a critical COVID-19 infection with mechanical ventilation, a prolonged course of recovery and the above-mentioned risk factors (arterial hypertension, age of 70, smoking history).

Recent literature states that long-term respiratory sequelae after COVID-19, including fibrotic lung disease and pulmonary vascular disease, are especially expected in patients after critical COVID-19 [5, 6]. In addition, arterial hypertension has recently been identified as a negative predictive value for worse COVID-19 outcome and elderly men are reported to have a strikingly higher COVID-19 mortality rate compared to younger individuals [7, 8]. To what extent a former nicotine abuse contributes to an adverse disease prognosis needs to be further investigated. However, in the largest study performed in China, the percentage of former smokers was higher in the group of hard or fatal COVID-19 courses of disease [9, 10].

In contrast to the functional limitations that are reported for patients at least three months after severe SARS-CoV-2 [11], this patient is thus a hopeful example that a very good functional status can be achieved under optimal conditions and rehabilitation. Spirometry and diffusion capacity normalized and assessment before lung surgery of pulmonary adenocarcinoma even displayed an oxygen uptake, allowing functional pulmonary resection up to a pneumectomy. In contrast, an Italian study showed low physical functioning and impaired performance of activities of daily living after hospitalization due to COVID-19 [12]. Further, a Dutch report on cardiopulmonary exercise training described cardiorespiratory fitness to be very poor with a median peak oxygen uptake of 15.0 ml O_2_/kg/min (57% of predicted values) in patients after COVID-19-induced mechanical ventilation [13].

The high level of athleticism of our patient may have contributed to the good outcome. However, during the first phase of the pandemic in northern Italy, the system was overwhelmed by the acute crisis, and care allocation decisions had to be taken in the absence of formal triage guidelines. This led to much distress on the part of individual clinicians and teams, who continuously had to make allocation decisions at the bedside. Therefore, prior to ICU treatment, the family of the patient was well aware of the concern that intensive medical treatment may not be advisable due to the patient’s age. Hence, this case supports the meanwhile established high consensus that age is no good discriminator to guide triage decisions in COVID-19 [14]. As we all know, outcome of COVID-19 disease is still precarious with patients suffering long term from disabilities. Therefore, this report is intended to be a motivation and, above all, a positive feedback for healthcare workers and especially ICU staff, who are committed to COVID-19 patients for weeks without knowing what quality of life can be regained.
